# Donor and Recipient Outcomes following Robotic-Assisted Laparoscopic Living Donor Nephrectomy: A Systematic Review

**DOI:** 10.1155/2019/1729138

**Published:** 2019-04-22

**Authors:** Massimiliano Creta, Armando Calogero, Caterina Sagnelli, Gaia Peluso, Paola Incollingo, Maria Candida, Gianluca Minieri, Nicola Longo, Ferdinando Fusco, Vincenzo Tammaro, Concetta Anna Dodaro, Francesco Mangiapia, Nicola Carlomagno

**Affiliations:** ^1^Department of Neurosciences, Human Reproduction and Odontostomatology, University Federico II, Naples, Italy; ^2^Department of Advanced Biomedical Sciences, University Federico II, Naples, Italy; ^3^Department of Mental Health and Public Medicine, University of Campania Luigi Vanvitelli, Italy

## Abstract

**Aims:**

We aimed to summarize available lines of evidence about intraoperative and postoperative donor outcomes following robotic-assisted laparoscopic donor nephrectomy (RALDN) as well as outcomes of graft and recipients.

**Methods:**

A systematic review of PubMed/Medline, ISI Web of Knowledge, and Scopus databases was performed in May 2018. The following search terms were combined: nephrectomy, robotic, and living donor. We included full papers that met the following criteria: original research; English language; human studies; enrolling patients undergoing RALDN.

**Results:**

Eighteen studies involving 910 patients were included in the final analysis. Mean overall operative and warm ischemia times ranged from 139 to 306 minutes and from 1.5 to 5.8 minutes, respectively. Mean estimated blood loss varied from 30 to 146 mL and the incidence of intraoperative complications ranged from 0% to 6.7%. Conversion rate varied from 0% to 5%. The mean hospital length of stay varied from 1 to 5.8 days and incidence of early postoperative complications varied from 0% to 15.7%. No donor mortality was observed. The incidence of delayed graft function was reported in 7 cases. The one- and 10-year graft loss rates were 1% and 22%, respectively.

**Conclusions:**

Based on preliminary data, RALDN appears as a safe and effective procedure.

## 1. Introduction

Renal transplantation is the treatment of choice for suitable patients with end-stage renal disease (ESRD) as it provides better outcomes in terms of life expectancy and quality of life than dialysis [1]. Kidney transplants from living donors confer advantages in terms of graft function and survival if we compared transplants from deceased donors [2]. Indeed, the elective nature of living donor transplantation offers the opportunity to have good quality grafts and to perform the procedure when the recipient is in an optimal clinical status. The main obstacle to living donation is the exposure of a healthy subject to the risks of a major surgical intervention. Therefore, efforts have been made to reduce complications and postoperative pain, achieve faster recovery, and minimize the surgical incisions. Laparoscopic donor nephrectomy (LDN) was first introduced in 1995 and is currently accepted as the gold standard for kidney procurement from living donors. This minimally invasive procedure has greatly enhanced living donation rates and in 2001 the number of living donors exceeded the number of cadaver donors [3]. To date, living donors account for most of the kidney donor pool in Western countries [4]. However, deceased donors still represent about 67.6% of transplanted kidneys [4]. In 2000, the US Food and Drug Administration approved the da Vinci Surgical System (Intuitive Surgical Inc.), a system that combines robotic techniques and computer imaging to enable microsurgery in a laparoscopic environment [5]. Advantages of the da Vinci Surgical System include the precision and instinctive movements of open surgery, an optimal ergonomic environment for the surgeon, and a 3-dimensional vision system that restores the hand-eye coordination lost in laparoscopic procedures [5–7]. The first worldwide robotic assisted laparoscopic donor nephrectomy (RALDN) was performed successfully at the University of Illinois at Chicago in 2000 by Horgan et al. [3]. Since then, the adoption of RALDN has increased worldwide and evidence about this procedure has slowly increased. The aim of the present review was to summarize available lines of evidence about intra- and postoperative donor outcomes following RALDN as well as outcomes of grafts and recipients.

## 2. Materials and Methods

We performed a systematic review using the Preferred Reporting Items for Systematic Reviews and Meta-Analyses Statement as a guideline in the development of the study protocol [19]. In May 2018 we used the National Library of Medicine PubMed search engine, the Scopus database, and the ISI Web of Knowledge official website to search for all published studies evaluating lines of evidence about donor and recipient outcomes following RALDN. The following search terms were combined: nephrectomy, robotic, and living donor. We included publications that met the following criteria: reporting original clinical studies; English language. Reference lists in relevant articles and reviews were also screened for additional studies. Abstracts (with no subsequent full-text publications) and unpublished studies were not considered. The quality of the randomized controlled trials was assessed using the Jadad score [20]. The following data were extracted from included studies: first author, year of publication, study design, sample size, study period, donors' age and sex, side of nephrectomy, surgical technique, control group, operative time (OT), console time (CT), warm ischemia time (WIT), estimated blood loss (EBL), conversion rate (CR), incidence, type and grade of intraoperative and postoperative complications, length of hospital stay (LOS), hemoglobin decrease, transfusion rate (TR), postoperative pain, duration of follow-up, last estimated glomerular filtration rate (eGFR), last creatinine, incidence of delayed graft function (DGF), duration of recipients' follow-up, recipients' last eGFR, recipients' last creatinine, graft survival, and recipients' complications.

## 3. Results

**Figure 1:**
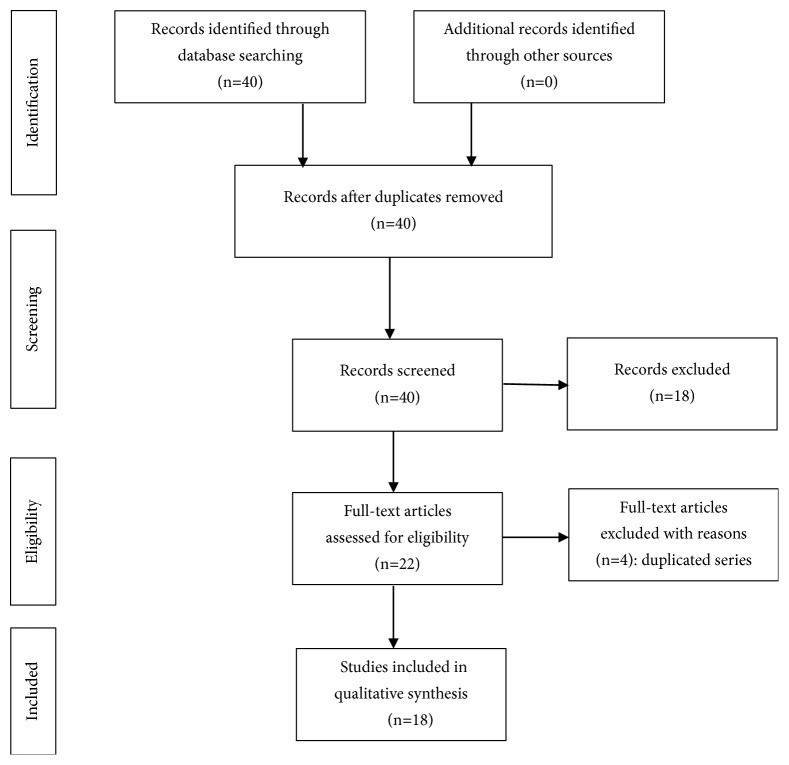
Flow diagram of the systematic review.

The search strategy revealed a total of 40 results. Screening of the titles and abstracts revealed 22 papers eligible for inclusion. Further assessment of eligibility, based on full-text papers, led to the exclusion of 4 papers. Finally, 18 studies involving a total of 910 patients who underwent RALDN from 2000 to 2018 were included in final analysis [2–18] (Figure 1). Specifically, 8 studies (44.4%) were retrospective observational, 4 (22.2%) were prospective observational, 1 (5.5%) was a randomized controlled trial, and 5 (27.7%) were case reports. The only randomized controlled trial was of low methodological quality (Jadad score =2). Eight studies (44.4%) had a control arm. The characteristics of the studies included are summarized in Table 1.

### 3.1. Intraoperative Outcomes

Table 2 summarizes RALDN-related intraoperative outcomes. Left kidneys were procured in 93.85% (n=854) of the donors.

### 3.2. Operative Times

Mean OT and mean CT ranged from 139 to 306 minutes and from 82 to 120 minutes, respectively. Three comparative studies found mean OT to be significantly longer in RALDN series with respect to LDN [4, 13, 16]. Mean OT of RALDN was also significantly longer than open donor nephrectomy (ODN) and hand-assisted retroperitoneoscopic donor nephrectomy (HARP) [4, 7, 13]. The relationship between vascular anatomy and OT is controversial. Gorodner et al. found that OT was significantly longer in patients with vascular anomalies with respect to those with normal vascular anatomy [8]. Similarly, Horgan et al. found OT to be significantly longer in patients with multiple renal arteries with respect to patients with normal anatomy [3]. Unlike previous authors, Janky et al. found no significant differences in terms of OT between donors with simple and complex vascular anatomy [13]. Interestingly, mean OT has been reported to significantly decrease with experience [3, 13]. In their study, Horgan et al. found a significant decrease in OT when their series was divided into three periods and the first 74 cases (201 minutes) were used for comparison with those in the second period (cases 75-144, 129 minutes) and third period (cases 145-214, 103 minutes) [3]. Similarly, Janki et al. found mean OT of RALDN procedures 1-19 to be significantly longer than RALDN procedures 40-59 (median OT of 240 and 172.5 minutes, respectively) [13]. In the study by Yang et al., the OT of RALDN approached that of LDN with each subsequent procedure over the course of the robotic cases and the standard OT of LDN was reached at the 22nd case [16].

### 3.3. Warm Ischemia Time

WIT ranged from <1.5 to 5.8 minutes. The impact of surgical technique on WIT is controversial. Two comparative studies found significantly longer WIT in patients undergoing RALDN with respect to those undergoing LDN [12, 16]. Two other comparative studies found WIT to be significantly longer in patients undergoing ODN and HARP [7, 13]. Unlike previous authors, Liu et al. and Janki et al. failed to find significant differences in terms of WIT between RALDN and LDN [1, 13]. The impact of vascular anatomy on WIT is controversial. Gorodner et al. found WIT to be significantly lower in patients with normal vascular anatomy with respect to patients with vascular anomalies [8]. Horgan et al. failed to find significant differences between patients with multiple renal arteries and patients with normal vascular anatomy in terms of WIT [3].

### 3.4. Intraoperative Complications

The incidence of intraoperative complications ranged from 0% to 6.7%. Bleeding was the most frequent complication and was reported in 11 patients (1.2%). Mean EBL varied from 30 mL to 146 mL. Janki et al. found EBL to be significantly lower in patients undergoing RALDN compared to HARP and LDN [13]. Serrano et al. found significantly lower EBL in patients undergoing RALDN compared to ODN, hand assisted LDN (HALDN) and LDN (80 mL, 296 mL, 91 mL, and 130 mL, respectively) with intraoperative transfusion rate of 0%, 0.3%, 0.5%, and 3% in RALDN, HALDN, LDN, and ODN, respectively (p<0.05) [4]. Other authors failed to find statistically significant differences between RALDN and LDN in terms of EBL [10, 13, 16]. One study found EBL to be significantly higher in patients with vascular abnormalities (107 mL vs 72 mL, p<0.05) [3].

### 3.5. Conversion Rates

CR varied from 0% to 5%. Overall, 14 cases (1.5%) of open conversion were reported. The reasons for conversion included inability to control bleeding from lumbar veins, bleeding of the stump of the renal artery, failure of the stapling device on the renal artery stump, and bleeding from renal vein laceration [3–5, 8, 10, 13]. In the study by Gorodner et al., all conversions occurred during the initial 100 cases [10]. Similarly, all the 4 conversions reported by Horgan et al. occurred in the first 74 cases [3].

### 3.6. Early (< 30 Days) Postoperative Donors' Outcomes

RALDN-related early postoperative donors' outcomes are summarized in Table 2.

### 3.7. Hospital Length of Stay

The mean hospital LOS ranged from 1 to 5.8 days. One comparative study found mean hospital LOS to be significantly shorter after RALDN compared to LDN [12]. Two other comparative studies found mean hospital LOS to be significantly shorter after RALDN compared to ODN [4, 7]. Five studies failed to find statistically significant differences between RALDN and LDN [1, 4, 10, 13, 16]. Cohen et al. found that mean LOS decreased with increasing RALDN experience [5]. Indeed, mean hospital LOS was 1.5 days, 2.3 days, and 2.0 days in the last 80 RALDN procedures, in the initial 20 RALDN, and in the last HALDN, respectively, with 20% of donors undergoing HALDN still in the hospital on postoperative day 3 compared to only 3.7% of donors in the last 80 RALDN procedures [5].

### 3.8. Postoperative Complications

The incidence of early postoperative complications ranged from 0% to 15.7%. In their study, Horgan et al. found that the postoperative complication rate dropped from 24% in the first 74 cases to a steady rate of 7% in the last part of their cohort [3]. Only 4 studies graded complications according to the Clavien Classification system by showing a high prevalence of Grades I and II complications [12, 13, 15, 16]. Comparative studies failed to find statistically significant differences between RALDN, ODN, LDN, and HARP in terms of postoperative complications [12, 13, 16]. No donor mortality was observed.

### 3.9. Postoperative Pain

Postoperative pain was investigated by three studies [2, 11, 12]. In the study by Bhattu et al., visual analogue scale scores for postoperative pain at 6, 24, and 48 hours as well as analgesic requirement were significantly lower after RALDN compared to LDN [12].

### 3.10. Late (>30 Days) Postoperative Donor Outcomes

Late postoperative donor outcomes were investigated by four studies [4, 9, 13, 16]. Mean follow-up ranged from 1.5 to 120 months (Table 2). Janky et al. failed to find statistically significant differences in terms of serum creatinine and eGFR at 3 months' follow-up after RALDN or LDN [13]. Yang et al. found similar results at one-year follow-up [16]. Serrano et al. found the incidence of ESRD to be close to 0% for all donors at 10 years' follow-up and this outcome was not influenced by surgical procedure (ODN, LDN, HA-LDN, and RALDN) [4]. The incidence of late complications was similar between RALDN and LND at one-year follow-up [16].

### 3.11. Recipient and Graft Outcomes

Table 3 summarizes results from studies evaluating recipient and graft outcomes relative to donors undergoing RALDN. Most of grafts functioned immediately after transplantation and DGF was reported in 7 cases. The incidence of DGF was not influenced by the surgical procedure adopted to procure the kidney (RALDN or LDN) [1]. Janki et al. failed to find significant differences in graft or recipient survival between RALDN and LDN as well as between RALDN and HARP at three-month follow-up [13]. In the study by Renoult et al. no significant differences emerged between recipients of donors undergoing RALDN and ODN [7]. Similarly, Yang et al. found no significant differences in terms of recipients' and grafts' outcomes at one-year follow-up regardless of the donor procedure [16]. In detail, there were no cases of graft failure or DGF in recipients of donors undergoing RALDN while there were three cases of graft failure and one case of DGF in patients who received kidneys from donors undergoing LDN [16]. eGFR was similar between recipients of donors undergoing RALDN and LDN at one-year follow-up [16]. In the study by Bhattu et al., eGFR at 7 days and at 1, 3, 6, and 9 months were similar among recipients of donors undergoing RALDN and LDN and no graft loss was observed in the two groups [12]. Indeed, each of the transplanted kidneys functioned correctly following surgery and none of the recipients required posttransplant dialysis [7]. Based on the measurement of the creatinine reduction ratio from posttransplantation day 1 to day 2, graft function improved more rapidly in the first two days after transplantation in the RALDN group with respect to ODN (42.6% vs 32.6%, p=0.01) and the mean estimated creatinine clearances at day 5 showed no differences between the two groups [7]. In the study by Serrano et al. the one-year graft loss rate for recipients whose living donor was in the ODN cohort was 9% versus the 2% in HA-LDN, 2% in P-LND, and 1% in RALDN cohorts [4]. The 10-year graft loss rate for recipients whose living donor was in the ODN, HA-LDN, P-LDN, and RALDN cohort was 27%, 23%, 20%, and 22%, respectively [4].

## 4. Discussion

Kidney transplant represents a common surgical intervention, with many cases performed yearly around the world [21, 22]. The procedure offers advantages over chronic dialysis in terms of quality of life and life expectancy. The number of patients requiring kidney transplantation increases with time and an improvement of the donation experience is strongly advocated [5, 16]. Unlike most surgical procedures, live donor nephrectomy is a unique, elective procedure, where a subject undergoes surgery for the sole benefit of another [16]. Therefore, it is of great importance to keep the morbidity and mortality of the procedure as low as possible [23]. Moreover, efforts should be made to procure the kidneys in optimal conditions for transplantation [23]. For many years, live donor nephrectomy was performed only with an open surgical approach and thereby many potential donors were reluctant to donate due to the morbidity associated with the procedure [23]. In 1995, Ratner et al. performed the first LDN at Johns Hopkins University of Baltimore [24]. LDN demonstrated several improvements over ODN such as decreased postoperative pain, decreased hospital LOS, faster recovery, and reduced perioperative blood loss [16]. Due to these advantages, LDN has become the standard of care and several modifications have been made to improve the technique [16]. The introduction of precise surgical robotic systems, like the da Vinci system, has expanded surgeons' ability to complete complex surgical tasks in a minimally invasive fashion. Some authors hypothesized that robotic assistance could result in a shorter and simpler learning curve for the procurement of kidneys from living donors and that it could enable an easier and more efficient management of complications [15]. By decreasing the learning curve for difficult surgical tasks, surgical robots may also expand the number of available surgeons for complex interventions as well as allow newer surgeons to quickly master these procedures [16]. In 2000, Horgan et al. performed for the first time a RALDN [25]. Since then, it has been adopted by several Institutions worldwide and the amount of evidence has progressively increased. To date, RALDN represents an evolving field. A new surgical technique should be compared against the gold standard. OT, EBL, incidence of complications, and conversion to open surgery are relevant intraoperative outcomes for most laparoscopic and robotic surgical procedures. The OT of LDN has been reported to range from 183 to 340 minutes [23]. Some authors have reported significantly longer OT with RALDN compared to LDN. Yang et al. hypothesized that the longer OT could be a result of their cautious, slower approach with RALDN due to their initial unfamiliarity with the procedure [16]. Interestingly, it has been demonstrated that OT associated with RALDN significantly decreases with experience and approaches the mean OT of LDN after few cases. Bleeding represents the most frequent intraoperative complication. However, two of the most recent comparative studies found EBL to be significantly lower during RALDN compared to LDN [4, 13]. Other studies found no significant difference between the two techniques in terms of EBL. It has been hypothesized that robot assistance may allow surgeons to dissect rapidly and efficiently and to control problematic bleedings more easily [7]. The reported frequency of open conversion during LDN ranges from 0% to 13% [4, 23]. The CR reported in the overall RALDN population analyzed in the present review is within the published ranges for LDN. Interestingly, most conversions occurred during the early phase of the learning curve. Giacomoni et al. hypothesized that the use of the robotic assistance can help to avoid the open conversion in cases of acute bleeding as it may facilitate the repair of vascular lesions [15]. WIT has traditionally represented a major concern during donor nephrectomy as it has been thought that any increase in this parameter would have translated into a poor graft function [23]. However, this notion has been disproved by various studies. WIT during LDN ranges between 95 and 300 seconds. Globally, WIT reported during RALDN is within published ranges for LDN. Some authors reported significantly longer WIT with RALDN compared to LDN probably related to the extraction that is performed by a second attending surgeon [16]. As RALDN is a relatively new procedure, the learning curve is a possible cause of differences observed in some intraoperative outcomes including OT and WIT [16]. Major advantages of RALDN over LDN are in the early postoperative period. Indeed, RALDN is characterized by lower postoperative pain and shorter hospital LOS. Bhattu et al. hypothesized that one of the possible reasons for less pain following robotic surgery is robotic arms, which are pivoted around the port site and moved at fixed remote center [12]. Consequently, there is less leverage and lesser pressure at port sites with subsequent lesser trauma to abdominal wall tissues around the port [12]. Some authors attributed the short LOS in the robotic-assisted program to reduced manipulation of the peritoneum, better identification of dissection planes, and limited energy use from cauterization leading to minimal inflammation and pain [5, 12]. Shorter LOS makes the RALDN procedure more convenient for the donor by allowing him to return as soon as possible to routine activities [5]. In a systematic review and meta-analysis of published work relative to 32,038 nephrectomies following different surgical techniques, Kortram et al. reported a global complication rate of 9.3% with complication rate after LDN being 23% [15, 26]. The present review demonstrates that the incidence of early postoperative complications is similar between RALDN and LDN. Interestingly, most of the complications occurred at the beginning of the learning curve. Unfortunately, the incidence of late postoperative complications was largely underinvestigated. Graft function and survival as well as recipient outcomes are of great importance when considering kidney donor procedures. Available lines of evidence demonstrate that RALDN does not adversely affect allograft function and survival. Although OT and WIT are longer in many RALDN experiences with respect to LDN, this seems not to translate into poorer graft function or recipient outcomes [16]. The incidences of DGF, graft function, and survival were similar between recipients whose living donor was in the RALDN and LDN cohorts in many studies. These data are coherent with the evidence that WIT up to 720 seconds do not correlate with graft function [16, 27]. Major limitations of current robotic systems are high costs and lack of haptic feedback [15, 28, 29]. However, daily use of the robot may reduce robotic costs mainly in a high-volume institute if the system is made available to multidisciplinary surgical teams [15]. The adoption of single-site robotic platforms has been described by some authors [17]. Early experience showed the safety of this approach but found that the technology added cost and complexity without tangible benefits [17]. Although the single port technology may decrease surgical invasiveness, its widespread adoption in the clinical practice will require the development of dedicated articulating instruments, energy, and stapling devices [17]. Potential limits of available literature must be acknowledged: available studies are few, often of low methodological quality, and with short follow-up. They enroll a small number of patients and often populations and surgical technique employed are different. Moreover, the quality of life after RALDN remains largely underinvestigated. Finally, the outcomes in specific populations, such as older living donors, need to be addressed [30, 31].

## 5. Conclusions

Available studies point out the feasibility and safety of RALDN. Although OT and WIT have been reported to be longer with respect to LDN in some studies, a progressive improvement with experience is evident. The procedure can provide potential advantages in terms of EBL, hospital LOS, and postoperative pain with respect to LDN. Graft and recipient outcomes are comparable to LDN. However, the technique is still in its infancy in many Institutions and available lines of evidence are still of poor quality. Consequently, these results should be interpreted with caution and role of RALDN needs further investigations.

## Figures and Tables

**Table 1:** Study characteristics.

Author	Year of publication	Study design	Sample size (n)	Study period	Age, years (mean)	Male: Female	Left: Right	Technique	Control group(s)
Gorodner (n.v.a.) [8]	2006	Retrospective	148	2000-2005	35	74:74	148:0	RALDN	-

Gorodner (v.a.) [8]	2006	Retrospective	61	2000-2005	35	27:34	61:0	RALDN	-

Renoult [7]	2006	Retrospective	13	2002-2004	39.3	3:10	12:1	RALDN	ODN

Hubert [6]	2007	Prospective	38	2002-2006	43	15:23	37:1	RALDN	-

Horgan [3]	2007	Prospective	214	2000-2006	36	110:104	214:0	Hand Assisted RALDN	-

Pietrabissa [9]	2010	Case report	1	2010	48	0:1	1:0	RALDN with transvaginal extraction of the kidney	-

Geffner [10]	2011	Retrospective	35	2007-2008	44.5	14:21	35:0	RALDN	LDN

Kaouk [11]	2012	Case report	1	2012	61	0:1	1:0	Transvaginal hybrid natural orifice transluminal robotic donor nephrectomy	-

Galvani [2]	2012	Case report	1	2012	21	0:1	1:0	SIRA	-

Liu [1]	2012	Retrospective	5	2005-2011	34.8	n.r.	0:5	RALDN	LDN

Cohen: Initial 20 cases [5]	2015	Retrospective	20	2009-2010	36.1	n.r.	20:0	RALDN	HA-LDN

Cohen: Last 80 cases [5]	2015	Retrospective	80	2010-2012	37.7	n.r.	59:21	RALDN	HA-LDN

Bhattu [12]	2015	Randomized controlled	15	2014-2015	46.4	2:13	6:9	RALDN	LDN

Serrano [4]	2016	Retrospective	94	2004-2014	40	36:58	88:6	RALDN	ODN, HA-LDN, LDN

Janki [13]	2016	Prospective	59	2009-2014	53.0	23:36	59:0	RALDN	LDN, HARP

Heredia [14]	2016	Case report	1	2016	34	0:1	0:1	Hand assisted RALDN	-

Giacomoni [15]	2017	Retrospective	98	2009-2016	53	29:69	88:10	RALDN	-

Yang [16]	2017	Retrospective	22	2011-2016	38.2	12:10	20:2	RALDN	LDN

Lamattina [17]	2018	Prospective	3	2015	52	0:3	3:0	Single-site robotic surgery	-

Barman [18]	2018	Case report	1	2018	30	1:0	1:0		-

HA-LDN: Hand Assisted Laparoscopic Donor Nephrectomy, HARP: Hand Assisted Retroperitoneoscopic Donor Nephrectomy, LDN: Laparoscopic Donor Nephrectomy, ODN: Open Donor Nephrectomy, n.r.: not reported, n.v.a.: normal vascular anatomy, SIRA: Single incision robotic assisted donor nephrectomy, and v.a.: vascular anomalies.

**Table 2:** RALDN associated intraoperative and postoperative outcomes.

Author	Intraoperative data							Early (< 30 days) postoperative data						Late (> 30 days) postoperative data			
	OT, min (mean)	CT, min	WIT, min	EBL mL	CR n (%)	Complications		LOS, days	Hb decrease	TR	Pain (VAS)	Complications		FU	Complication type	Last eGFR	Last creatinine
						n (%)	Type (n)					n (%)	type (CDC) (n)				
		(mean)	(mean)	(mean)				(mean)	(g/dL)	n (%)	mean			(mo)	(CDC) (n)	(mL/min/ 1.73m^2^)	(mg/dL)
Gorodner (n.v.a.) [8]	146	n.r.	1.5	76	3 (2.0)	1 (0,6)	Bleeding	2	n.r.	0 (0)	n.r.	11 (7.4)	Pneumonia (n=2) Pancreatitis (n=1) Superficial wound infection (n=8)	n.r.	n.r.	n.r.	n.r.
Gorodner (v.a.) [8]	158	n.r.	1.7	107	1 (1.6)			2	n.r.	0 (0)	n.r.			n.r.	n.r.	n.r.	n.r.

Renoult [7]	185.5*∗*	n.r.	7.1*∗*	n.r.	0 (0)	0 (0)		5.8*∗∗*	-0,9	0 (0)	n.r.	1 (7.6)	Deep vein thrombosis (n=1)	n.r.	n.r.	n.r.	n.r.

Hubert [6]	181	n.r.	5.8	n.r.	0 (0)	0 (0)		5.5	-0.8	0 (0)	n.r.	6 (15.7)	Acute pyelonephritis (n=2) Deep vein thrombosis (n=1) Pulmonary embolism (n=1) Wound infection (n=1) Urinary tract infection (n=1)	n.r.	n.r.	n.r.	n.r.

Horgan [3]	150	n.r	1.6	82	4 (1.8)	4 (1.8)	Bleeding	2	n.r.	n.r.	n.r.	24 (11.2)	Pneumonia (n=1) Pancreatitis (n=1) Wound infection (n=10) Ileus (n=11) Ventral hernia (n=1)	n.r.	n.r.	n.r.	n.r.

Pietrabissa [9]	215	95	3,15	<50	0 (0)	0 (0)		1	n.r.	0 (0)	n.r.	0 (0)	0	1.5	n.r.	65	n.r.

Geffner [10]	149	n.r.	n.r.	146	1 (2.9)	1 (2.8)	Bleeding	3.2			n.r.	1 (2.8)	Acute urinary retention	n.r.	n.r.	n.r.	n.r.

Kaouk [11]	240	120	5,46	n.r.	0 (0)	0 (0)		2	n.r.	0 (0)	24h:3/10	0 (0)	0	n.r.	n.r.	n.r.	n.r.

Galvani [2]	150	n.r.	3,3	75	0 (0)	0 (0)		3	n.r.	0 (0)	24h: 6/10 72 h: 2/10	0 (0)	0	n.r.	n.r.	n.r.	n.r.

Liu [1]	218	n.r.	3.8	30	0 (0)	0 (0)		3.6	n.r	0 (0)	n.r.	n.r.	n.r.	n.r.	n.r.	n.r.	n.r.

Cohen: Initial 20 cases [5]	149	n.r.	n.r.	n.r.	1 (5)	1 (5)	Bleeding	2.3	n.r.	0 (0)	n.r.	2 (10)	Deep vein thrombosis (n=1) Bleeding (n=1)	n.r.	n.r.	n.r.	n.r.

Cohen: Last 80 cases [5]	139	n.r.	n.r.	n.r.	0 (0)	1 (1.2)	Tear in the mesocolon	1.5	n.r.	1 (1.2)	n.r.	2 (2.5)	Nausea (n=1) Bleeding (n=1)	n.r.	n.r.	n.r.	n.r.

Bhattu [12]	156.6	n.r.	5.3*∗*	n.r.	0 (0)	0 (0)		3*∗∗*	-0.8		6h:4.3/10*∗∗* 24h:2.7/10*∗∗* 48h:1.4/10*∗∗*	2 (13.3)	n.r. (I) (n=2)	n.r.	n.r.	n.r.	n.r.

Serrano [4]	306*∗*	n.r	n.r.	80*∗∗*	3 (3.1)	3 (3.1)	n.r.	3*∗∗*	n.r	0 (0)	n.r.	8 (8.5)	Infection (n=1) Ileus (n=3) Urinary tract infection (n=4)	120	Incisional hernia (n=1)	n.r.	1.01

Janki [13]	205.0*∗*	n.r.	4.0*∗*	100*∗∗*	1 (1.6)	4 (6.7)	Kidney torsion (n=1), Stapler malfunction (n=1), bleeding (n=2)	2.0	n.r.	0 (0)	n.r.	5 (8.4)	Wound infection (I) (n=1) Subcutaneous hematoma (I) (n=1) Seroma of the trocar incision (I) (n=1) Urinary tract infection (II) (n=1) Chylous leakage (IIIA) (n=1)	3	Nausea-dizziness (I) (n=1)	54.0	1.07

Heredia [14]	170	n.r.	<1.5	n.r.	0 (0)	0 (0)		3	n.r.	n.r.	n.r.	0 (0)	0	n.r.	n.r.	n.r.	n.r.

Giacomoni [15]	239	111	n.r.	n.r.	n.r.	3 (3.0)	Bleeding (n=2) Pneumothorax (n=1)	4,5	n.r.	2 (2.0)	n.r.	13 (13.2)	Seroma (I) (n=5) Rhabdomyolysis (II) (n=1) Subocclusion (II) (n=1) Paralytic ileus (II) (n=1) Hypertensive crisis (II) (n=1) Oxygen desaturation (II) (n=1) Postoperative anemia (II) (n=1) Chylous collection (III) (n=2)	n.r.	n.r.	n.r.	n.r.

Yang [16]	192.3*∗*	n.r	3.4*∗*	55.9	n.r.	n.r.		2.5	-1.8	0 (0)	n.r.	1 (4.5)	Chylous leakage (II) (n=1)	12	Nausea (I) (n=1)	60.6	1.2

Lamattina [17]	262	82	n.r.	77	0 (0)	0 (0)		2	n.r	n.r	n.r.	n.r.	n.r.	n.r.	n.r.	n.r.	n.r.

Barman [18]	215	n.r.	5	50	n.r.	n.r.		n.r.	n.r.	n.r.	n.r.	n.r.	n.r.	n.r.	n.r.	n.r.	n.r.

*∗*: significantly higher with respect to control group, *∗∗*: significantly lower with respect to control group, CDC: Clavien Dindo classification, CR: conversion rate, CT: console time, EBL: estimated blood loss, eGFR: estimated glomerular filtration rate, FU: follow-up, LOS: length of stay, mo: months, n.r.: not reported, n.v.a.: normal vascular anatomy, OT: operative time, TR: transfusion rate, v.a.: vascular anomalies, VAS: Visual Analog Scale, and WIT: warm ischemia time.

**Table 3:** Graft and recipient outcomes.

Author	DGF n (%)	Follow-Up (mo)	Serum creatine, mg/dL, mean (Follow-up)	eGFR mL/min/1.73m^2^ (Follow-up)	Graft survival, % (Follow-up)	Complications, type (n)
Gorodner (n.v.a.) [8]	0 (0)	12	1.4 (6 mo)	n.r.	96.6 (12 mo)	Vascular thrombosis (2) Pyeloureteral junction stricture (1)

Gorodner (v.a.) [8]	0 (0)	12	1.4 (6 mo)	n.r.	96 (12 mo)	0

Renoult [7]	0 (0)	1	n.r.	n.r.	100 (1mo)	0

Hubert [6]	0 (0)	1	n.r.	n.r.	100 (1 mo)	Temporary pyeloureteral dilatation (1)

Horgan [3]	0 (0)	12	1.4 (6 mo)	n.r.	98 (12 mo)	Renal artery thrombosis (1) Acute rejection (1) Abnormal kidney anatomy (1)

Pietrabissa [9]	0 (0)	2	n.r.	n.r.	100 (2 mo)	0

Geffner [10]	n.r	120	n.r.	n.r.	97.1 (12 mo)	Graft rejection due to lymphoma (1)

Kaouk [11]	1 (100)	1	1.1 (1 mo)	67 (1 mo)	100 (1 mo)	Acute rejection (1)

Galvani [2]	0 (0)	3	0.8 (3 mo)	n.r.	100 (3 mo)	0

Liu [1]	1 (20)	12	1.4 (12 mo)	60.8 (12 mo) *∗*	n.r.	n.r.

Cohen (Initial 20 cases) [5]	n.r.	1	2	n.r.	n.r.	n.r.

Cohen (last 80 cases) [5]	n.r.	1	1.6	n.r.	n.r.	n.r.

Bhattu [12]	0 (0)	9	n.r.	68.7 (9 mo)	100 (9 mo)	Acute tubular necrosis (1) Recurrence of focal segmental glomerulosclerosis (1)

Serrano [4]	4 (4.2)	120	1.4 (12 mo)	n.r.	99 (12 mo) 89 (60 mo) 78 (120 mo)	Acute rejection (28)

Janki [13]	n.r.	3	n.r.	n.r.	94.9 (3 mo)	n.r.

Heredia [14]	n.r.	n.r.	n.r.	n.r.	n.r.	n.r.

Giacomoni [15]	1 (1.0)	n.r.	n.r.	n.r.	n.r.	Intraoperative venous thrombosis (1)

Yang [16]	0	120	1.2 (12 mo)	70.6 (12 mo)	0 (120 mo)	n.r.

*∗*: statistically significant higher if compared to control group, DGF: delayed graft function, eGFR: estimated glomerular filtration rate, mo: months, and n.r.: not reported.
